# Alternatively Activated Mononuclear Phagocytes from the Skin Site of Infection and the Impact of IL-4Rα Signalling on CD4^+^T Cell Survival in Draining Lymph Nodes after Repeated Exposure to *Schistosoma mansoni* Cercariae

**DOI:** 10.1371/journal.pntd.0004911

**Published:** 2016-08-09

**Authors:** Catriona T. Prendergast, David E. Sanin, Adrian P. Mountford

**Affiliations:** Centre for Immunology and Infection, Department of Biology, University of York, York, United Kingdom; Uniformed Services University, UNITED STATES

## Abstract

In a murine model of repeated exposure of the skin to infective *Schistosoma mansoni* cercariae, events leading to the priming of CD4 cells in the skin draining lymph nodes were examined. The dermal exudate cell (DEC) population recovered from repeatedly (4x) exposed skin contained an influx of mononuclear phagocytes comprising three distinct populations according to their differential expression of F4/80 and MHC-II. As determined by gene expression analysis, all three DEC populations (F4/80^-^MHC-II^high^, F4/80^+^MHC-II^high^, F4/80^+^MHC-II^int^) exhibited major up-regulation of genes associated with alternative activation. The gene encoding RELMα (hallmark of alternatively activated cells) was highly up-regulated in all three DEC populations. However, in 4x infected mice deficient in RELMα, there was no change in the extent of inflammation at the skin infection site compared to 4x infected wild-type cohorts, nor was there a difference in the abundance of different mononuclear phagocyte DEC populations. The absence of RELMα resulted in greater numbers of CD4^+^ cells in the skin draining lymph nodes (sdLN) of 4x infected mice, although they remained hypo-responsive. Using mice deficient for IL-4Rα, in which alternative activation is compromised, we show that after repeated schistosome infection, levels of regulatory IL-10 in the skin were reduced, accompanied by increased numbers of MHC-II^high^ cells and CD4^+^ T cells in the skin. There were also increased numbers of CD4^+^ T cells in the sdLN in the absence of IL-4Rα compared to cells from singly infected mice. Although their ability to proliferate was still compromised, increased cellularity of sdLN from 4x IL-4RαKO mice correlated with reduced expression of Fas/FasL, resulting in decreased apoptosis and cell death but increased numbers of viable CD4^+^ T cells. This study highlights a mechanism through which IL-4Rα may regulate the immune system through the induction of IL-10 and regulation of Fas/FasL mediated cell death.

## Author Summary

In areas endemic for schistosomiasis, repeated exposure to infective cercariae is a frequent occurrence, and repeated exposure of murine skin to *Schistosoma mansoni* resulted in CD4^+^ T cells becoming hypo-responsive. Here potential contributory mechanisms were investigated. In the skin infection site, three mononuclear phagocyte populations were identified (tissue macrophages, dendritic cells, and macrophages) which exhibited up-regulation of genes associated with alternative activation, in particular the gene encoding RELMα. However, in repeatedly infected mice deficient in RELMα, there was no change in the abundance of mononuclear phagocytes in the skin, and CD4^+^ cells in the skin draining lymph nodes remained hypo-responsive. In mice deficient for IL-4Rα, required for alternative activation, levels of dermal regulatory IL-10 were reduced and there was an increase in the abundance of antigen presenting MHC-II^high^ cells, which was accompanied by increased numbers of CD4^+^ T cells. Although the absence of IL-4Rα did not translate into increased CD4^+^ cell responsiveness, they exhibited lower expression of Fas/FasL, resulting in decreased apoptosis/cell death and increased cell viability. This study highlights a mechanism through which IL-4Rα may regulate the immune system through the induction of IL-10 and regulation of Fas/FasL mediated cell death.

## Introduction

Schistosomiasis is a debilitating disease that develops following percutaneous infection with the parasitic helminth *Schistosoma spp* [1, 2]. The disease affects approximately 230 million people worldwide with infection occurring when the skin is exposed to the free-swimming tissue invasive cercariae [3]. Since the infective stage of the parasite is often present in water used for domestic purposes, individuals living in areas endemic to schistosomiasis are at risk of repeated infections.

In order to investigate the effect of repeated infection with schistosome cercariae on the immune response, we developed an experimental model whereby mice were exposed via their pinnae once (1x), or repeatedly (4x), to doses of infective *S*. *mansoni* cercariae [4]. It was found that after 4x, compared to 1x, exposures there were major changes in the cell populations within the skin site of infection such that eosinophils, macrophages, dendritic cells (DCs), neutrophils, mast cells, CD4^+^ T cells and keratinocytes were all increased after 4x infections [4–7]. Moreover, repeated infections resulted in the development of CD4^+^ T cell hypo-responsiveness in the skin-draining lymph nodes (sdLN), as well as decreased immunopathology in the liver generated in response to eggs released by adult worms [4]. CD4^+^ T cells in the sdLN from 4x mice had reduced ability to proliferate and secrete cytokines in response to larval schistosome antigens, and this was shown to be IL-10 dependent [6, 8].

Exposure of the skin to repeated doses of schistosome cercariae caused major changes in the local cytokine environment, particularly the levels of IL-4, IL-13 and IL-10, and it was proposed that immune responses in the skin involved mononuclear phagocytes that were alternatively activated [4]. The term alternative activation conventionally describes macrophages under the influence of IL-4 and IL-13 [9–12]. Alternatively activated macrophages have been given the term M2, or subgroupings thereof M2a-c [13], whilst the term alternative activation has also been used in the context of DCs [14]. Parasitic helminth infections are often associated with the development of alternatively activated cells and polarized Th2-type immune responses [15]. Together, these cell populations are linked to the development of wound healing, which accompanies the response to tissue invasive helminths [16, 17]. Therefore, it is likely that there will be substantial wound healing following the repeated percutaneous invasion of the skin by schistosome cercariae.

In the current study, we set out to investigate mononuclear phagocyte cell populations in the skin infection site following repeated exposure to schistosome larvae, and found evidence for a gene expression profile associated with alternative activation. Subsequently, we focused our investigation upon the impact of IL-4Rα which is a receptor for both IL-4 and IL-13 essential for the alternative activation of macrophages [9, 10], and Resistin-like molecule α (RELMα) which is a key marker of alternative activation and is abundant during Th2 immune responses in allergic lung inflammation and helminth infection [18–21]. Consequently, we hypothesized that IL-4Rα and RELMα might play an important role in the response following repeated exposures to infectious *S*. *mansoni* cercariae.

## Materials and Methods

### Ethics statement

Experiments were carried out in accordance with the United Kingdom Animals Scientific Procedures Act 1986 and with the approval of the University of York Ethics Committee (PPL 60/4340).

### Mice

C57BL/6 wild-type (WT), IL-4Rα deficient (Il-4rα^-/-^; IL-4RαKO) [22], and Resistin-like molecule α deficient (*Retlnα*^-/-^; RelmαKO) [23], mice were bred and housed at the University of York. Both IL-4RαKO and RelmαKO mice on C57BL/6 background were kind gifts from Professor Judith Allen, University of Edinburgh. Age and sex-matched mice (between 6 and 10 weeks) were used for all experiments.

### *S*. *mansoni* parasites and skin infection

The life cycle of a Puerto Rican strain of *S*. *mansoni* was maintained at the University of York. Both pinnae of mice were percutaneously exposed to 150 *S*. *mansoni* cercariae either once only (1x), or four times in total (4x) on a once-a-week basis between day 0 to day 21, as described previously [4, 24]. Pinnae and skin-draining lymph nodes (sdLN; auricular lymph nodes) were harvested 4 days after the final infection. Skin infection via the pinnae results in a 50% penetration rate [24], therefore a dose of approximately 75 cercariae per pinna is achieved. Pinnae inflammation was measured using a dial gauge micrometer (Mitutoyo, Japan).

### Isolation of dermal exudate cells (DEC)

Pinnae were collected and briefly exposed to 70% ethanol to sterilize before being air-dried. They were then split along the central cartilage into two halves, and floated on the surface of complete RPMI media (RPMI-1640 (Gibco, Paisley, UK) containing 10% heat inactivated FCS (Biosera, Uckfield, UK), 2 mM L-Glutamine, 1% Pen/Strep (both Gibco) and 50 μM 2-mercaptoethanol (Sigma-Aldrich, Gillingham, UK) overnight at 37°C 5% CO_2_, to obtain the dermal exudate cells (DEC) as previously described [4, 24, 25]. After overnight *in vitro* culture, the tissue samples were discarded, while the culture supernatants were spun at 1000 x*g* for 7 minutes at 4°C to recover the DEC. The skin biopsy culture supernatants were frozen at -20°C prior to subsequent analysis by ELISA. DEC were re-suspended in complete RPMI, numbers determined by trypan blue exclusion, and then subjected to either analysis by flow cytometry, or separated into groups by fluorescence-activated cell sorting (FACS).

### Cytokine analysis by ELISA

The amounts of released IL-4, IL-10, IL-12p40 present in the skin biopsy culture supernatants were determined using DuoSet enzyme-linked immunosorbent assay (ELISA) kits (R&D Systems, Abingdon, UK).

### *In vivo* lymph node cell proliferation

Mice received 1 mg bromodeoxyuridine (BrdU; Sigma-Aldrich) via daily intraperitoneal (i.p.) injection for the final 4 days prior to harvest of the sdLN in order to determine *in vivo* CD4^+^ cell proliferation [8]. Cells recovered from the sdLN were initially blocked with anti-CD16/32 monoclonal antibodies (mAbs; eBioscience, Hatfield, UK,) in goat serum (Sigma-Aldrich), and later labelled using anti-CD3 and anti-CD4 mAbs (both eBioscience) in PBS supplemented with 1% FCS (FACS Buffer). Cells were then washed in FACS Buffer, incubated in 1x Fixation/Permeabilization buffer (eBioscience) for one hour at 4°C, washed again, and then incubated at 37°C in 100 μg DNase (Sigma-Aldrich) for 1 hour. After a final wash in FACS buffer, cells were labelled for 45 minutes at room temperature with anti-BrdU mAb, or rat IgG1 isotype control mAb (both eBioscience), in 1x permeabilization buffer, according to the manufacturer’s instructions.

### Flow cytometry

DEC were first incubated using Fixable Live/Dead Aqua stain (Life Technologies, Paisley, UK), blocked with anti-CD16/32 mAbs (eBioscience) in goat serum (Sigma Aldrich), and subsequently labeled with the following mAbs conjugated to fluorescent labels: anti-CD45, anti-F4/80, anti-MHC-II (IA-IE), anti-Fas, anti-FasL, anti-CD3 and anti-CD4 (all eBioscience). Flow cytometry data was acquired on the Cyan ADP, or the BD LSR Fortessa analyzer (Beckman Coulter, London, UK). Data was analyzed using FlowJo Software v7.6.5 (Tree Star Inc, Oregon Bio, Oregon US).

### Annexin V assay

After surface staining for anti-CD3 and anti-CD4, sdLN cells were washed in cold PBS supplemented with 1x annexin V binding buffer (eBioscience), and incubated for 15 minutes with anti-annexin V FITC at room temperature. Cells were then washed in annexin V binding buffer and resuspended for analysis in annexin V binding buffer. Propidium iodide (PI) (eBioscience) was added directly before acquiring the data.

### FACS-sorting of DEC and microarray analysis

DEC obtained from infected pinnae in three independent experiments (12–18 mice each) recovered on day 4 after infection with *S*. *mansoni* cercariae were pooled and labelled with anti-MHC-II (IA-IE) (clone # M5/114) and anti-F4/80 (clone # BM8) mAbs. Cell populations which were F4/80^+^MHC-II^high^, F4/80^+^MHC-II^lo^, or F4/80^-^MHC-II^high^ were recovered by FACS (MoFlo Astrios, Beckman Coulter) and RNA extracted from sorted DEC populations using TRIzol (Life Technologies). RNA was quantified using a Nanodrop (Thermo Scientific, Waltham, USA) and quality checked using a Bioanalyzer (Agilent Technologies, Santa Clara, USA). Staff at the Technology Facility at the University of York, York, UK, prepared and carried out microarray analysis of purified RNA, including sample labelling and hybridisation, using the Agilent SurePrint system (Agilent Technologies).

### Bioinformatics

GeneSpring (Agilent Technologies) was used to normalize microarray data, calculate fold differences, prepare dendrograms of cell populations and establish significant (p< 0.05) differentially expressed genes.

### Statistics

Statistical analyses were performed using a one-way analysis of variance (ANOVA) and Tukey’s multiple comparisons test using GraphPad Prism v6 software (GraphPad Software Inc, San Diego, California. USA).

## Results

### Genes associated with alternative activation were highly up-regulated in DEC recovered from the skin after repeated exposures to *S*. *mansoni* cercariae

**Fig 1:**
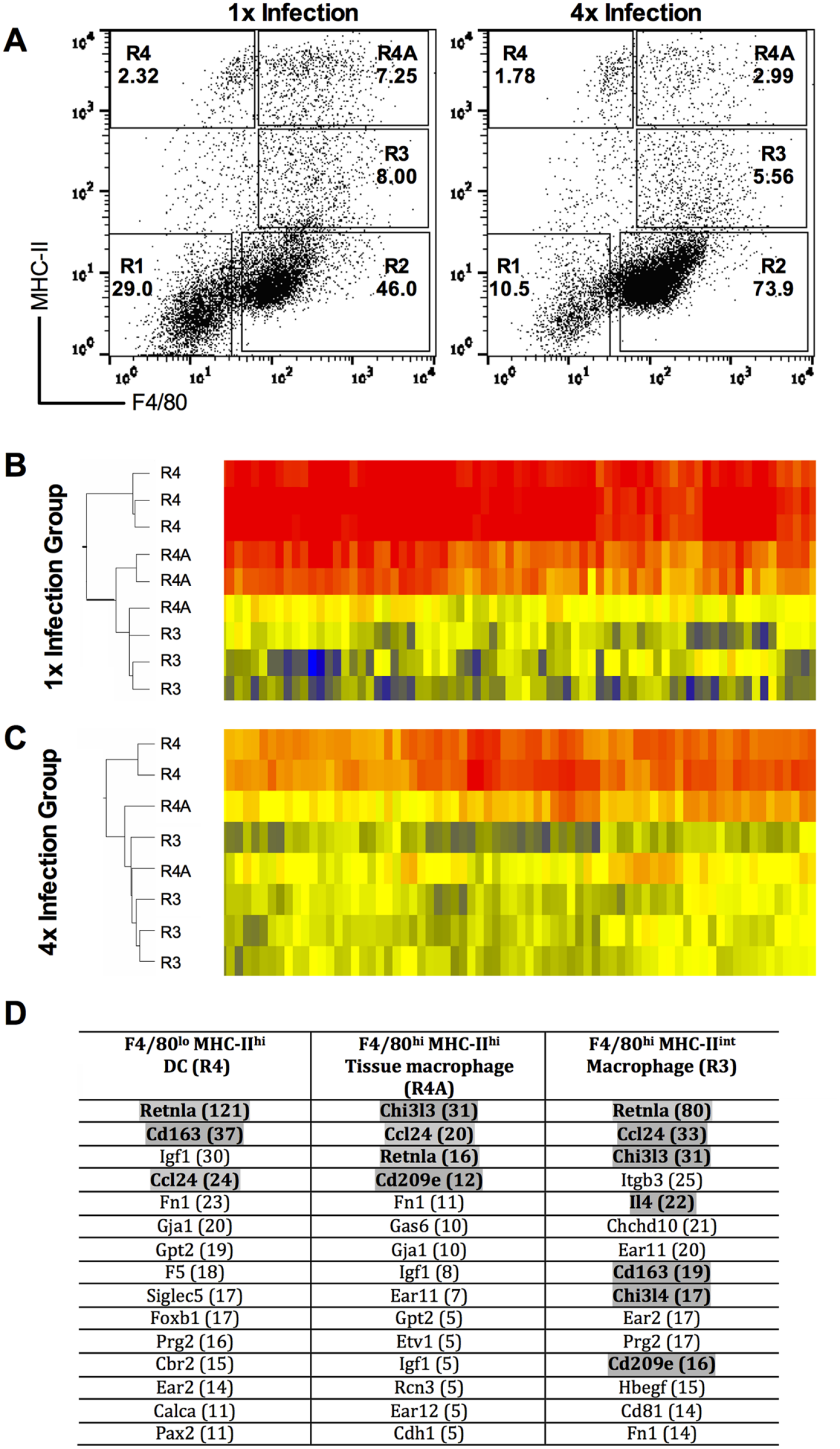
Genes related to alternative activation cell phenotype are up-regulated in dermal exudate cells (DEC) populations after repeated infection. **A.** Representative flow cytometry dot plots for DEC showing the distribution of cells based upon their expression of F4/80 and MHC-II. Cells were gated into R4, R4A and R3 populations recovered from 1x (left) and 4x (right) infected mice. Values in bold are the percent positive cells found within each gate expressed as a proportion of the total DEC recovered. Cells were sorted by fluorescent activated cell sorting (FACS), and RNA from the sorted R4, R4A and R3 cell populations was applied to microarray analysis. Heat maps showing the clustering of genes within each sorted DEC population for three biological replicates recovered after **B.** a single (1x) infection, and **C.** repeated (4x) infections. **D.** Identity of the top 15 up-regulated genes in each population after 4x compared to 1x infection. Number in brackets represents the fold up-regulation of that gene in the sorted cell population from 4x infected mice. Grey shaded genes represent those that are associated with alternative activation.

Dermal exudate cells (DEC) were recovered from *in vitro* cultured skin biopsies after exposure to either a 1x dose, or 4x doses, of *S*. *mansoni* cercariae in order to characterize genes that were differentially regulated four days after the final infection. Cells which were F4/80^+^MHC-II^-^, shown to be SiglecF^+^ eosinophils [4, 12] were excluded from the analysis as we previously showed that the absence of these cells had no effect on the development of CD4^+^ cell hypo-responsiveness in the sdLN [7]. Therefore, our microarray analysis focused upon cells of the mononuclear phagocyte system (i.e. DCs and macrophages). These cells distributed into three discrete populations: F4/80^-^MHC-II^high^ cells (denoted ‘R4’) were classed as DCs, F4/80^+^MHC-II^high^ cells (denoted ‘R4A’) were termed tissue resident macrophages, whilst F4/80^+^MHC-II^int^ cells (denoted ‘R3’) were classified as macrophages [6] as shown in Fig 1A.

Clustering analysis of microarray data obtained from biological replicates of the three sorted DEC populations is shown as dendrograms with corresponding selected heat maps for cells from 1x and 4x infected groups of mice (Fig 1B and 1C). The heat maps displayed highlight a section from the start of the entire microarray heat map, whilst the dendrograms are based upon analysis of all identified genes to yield an overall comparison. This revealed clear clustering patterns within each of the defined cell populations (i.e. R4, R4A, and R3) validating our gating strategy. Based on the dendrogram analysis of the microarray data from the 1x and 4x DEC populations, gene expression profiles within the R4A tissue resident macrophages were more closely associated to R3 macrophages than to R4 DCs (Fig 1B and 1C). Transcriptional distinctions between the R4A and R3 populations were reduced after 4x infection (Fig 1C).

Many identified genes found in the three sorted DEC populations were differentially up-regulated in the 4x compared to the 1x samples, and were linked to alternative activation (e.g. *retnla*, *chi3l3*, *chi3l4*, *ccl24*, *cd209* and *cd163* [11, 26–28]) (Fig 1D). *Retnla* (encoding RELMα) was one of the most highly up-regulated genes in 4x DCs (R4; x121-fold), 4x tissue macrophages (R4A; x16-fold) and 4x macrophages (R3; x80-fold), compared to their 1x counterpart cell populations. Similarly, *chi3l3* was up-regulated in both 4x tissue macrophages (R4A; x31-fold) and 4x macrophages (R3; x31-fold), whilst *ccl24* was up-regulated in all three cell populations 24-33-fold. The gene for IL-4 was up-regulated in 4x macrophages (R3; x22-fold), whilst *il4ra* was also up-regulated in these cells (R3; x6-fold). Other genes which featured in the top 20 up-regulated genes included those associated with tissue destruction/wound healing such as *igf1* (x8-30-fold) and *fn1* (x14-23-fold).

### Inflammation of the skin infection site in the absence of IL-4Rα and RELMα following 4x schistosome infection

**Fig 2:**
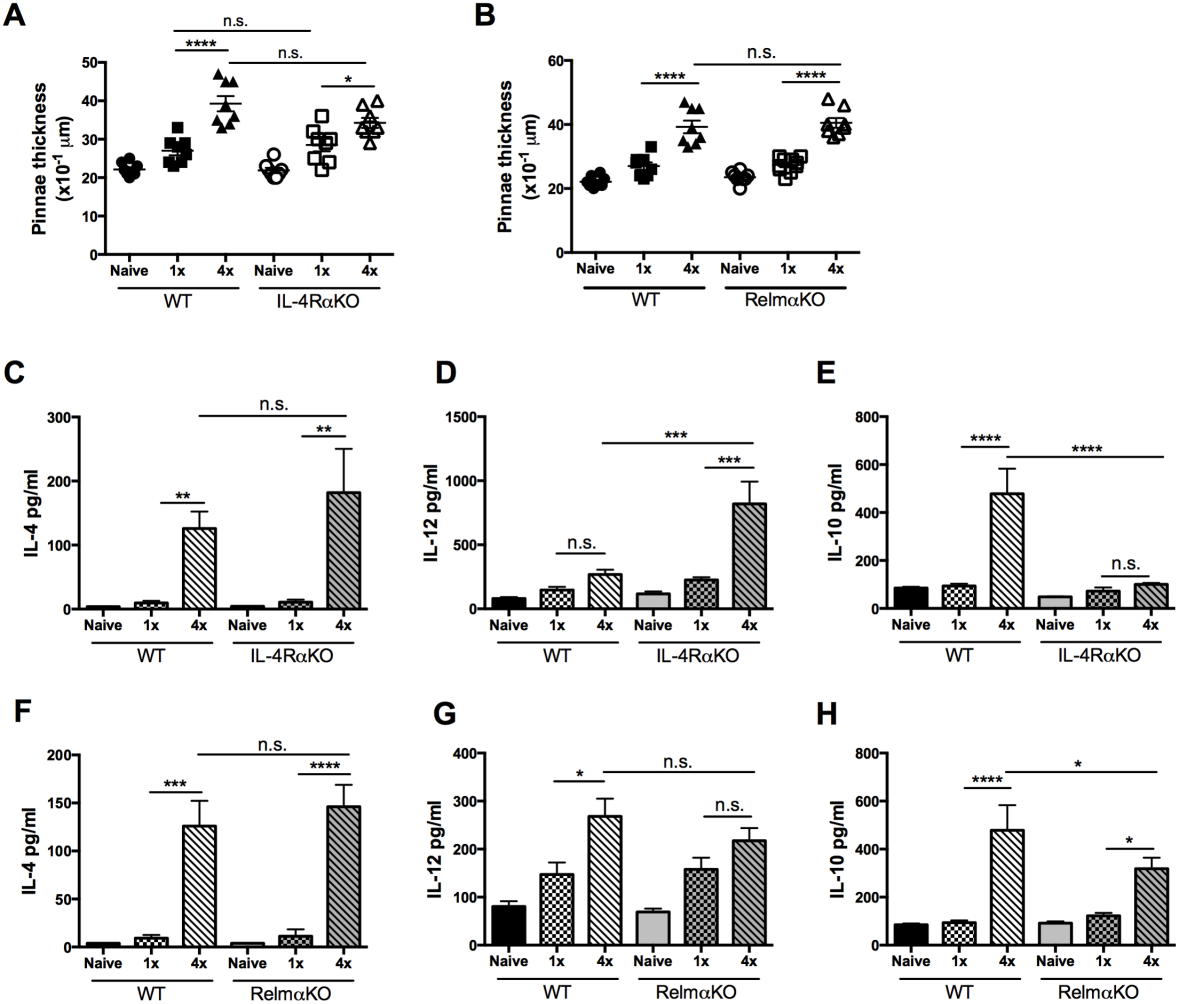
Inflammation of the skin infection site in the absence of IL-4Rα and RELMα following 4x schistosome infection. **A.** Pinnae thickness in naïve, 1x and 4x infected WT and IL-4RαKO mice and, **B.** in naïve, 1x and 4x infected WT and RelmαKO mice on day 4 after the final infection. Symbols are values for individual mice; horizontal bars are means ±SEM (n = 4–5 mice). **C-H.** Production of IL-4, IL-12p40 and IL-10 by skin biopsies (cultured in the absence of exogenous parasite antigen) from groups of WT, IL-4RαKO and RelmαKO mice. Cytokine production in the overnight culture supernatants was determined by ELISA (pg/ml); n = 4–5 mice per group. * = p<0.05; ** = p<0.01; *** = p<0.001, **** = p<0.0001, n.s. = p>0.05 as determined by ANOVA and Tukey’s post test analysis.

Given the significant changes in gene expression associated with IL-4Rα and RELMα, their roles in the early stage immune response were investigated using *Il-4rα*^*-/-*^, or *Retlnα*^*-/-*^ mice. After 4x exposures to infective *S*. *mansoni* cercariae, pinnae thickness of WT mice, as well as those deficient in IL-4Rα, was significantly increased compared to 1x exposure (Fig 2A; p<0.0001 and p<0.05 respectively). However, the thickness of the pinnae infection site was comparable between WT and IL-4RαKO mice, irrespective of the infection regime (Fig 2A; p>0.05). Similarly, whilst RelmαKO mice exposed to 4x doses of cercariae displayed significantly enhanced levels of skin inflammation compared to naive and 1x RelmαKO animals (all p<0.0001), the levels of inflammation were comparable with those of their 4x WT cohorts (Fig 2B; all p>0.05).

Increased levels of IL-4, IL-12p40 and IL-10 released by skin biopsies cultured *in vitro* in the absence of added antigen were detected in the pinnae of 4x compared to 1x WT mice (Fig 2C–2H; p<0.05–0.0001). In the absence of IL-4Rα, the levels of IL-4 in 4x IL-4RαKO mice were comparable to those in 4x WT samples (Fig 2C), on the other hand, the levels of pro-inflammatory IL-12p40 in 4x IL-4RαKO mice were significantly increased compared to 4x WT samples (Fig 2D; p<0.001). The levels of regulatory IL-10 were low in 4x IL-4RαKO mice (Fig 2E), resulting in a significant reduction in the quantities of IL-10 being released compared to 4x WT mice (Fig 2E; p<0.0001). The absence of RELMα had no effect on the production of IL-4, or IL-12p40 in 1x and 4x mice compared to their WT cohorts (Fig 2F and 2G; p>0.05), however similar to 4x IL-4RαKO mice, IL-10 production was significantly lower in 4x RelmαKO skin compared to 4x WT skin (Fig 2H; p<0.05). Therefore, although IL-4RαKO and RelmαKO mice produce similar amounts of IL-4 after 1x and 4x infections, IL-4RαKO mice have a more pro-inflammatory phenotype (i.e. increased IL-12p40) and both KO strains had decreased IL-10 production compared to WT mice.

### The absence of IL-4Rα, but not RELMα, increases the abundance of mononuclear phagocytes recovered from the skin

**Fig 3:**
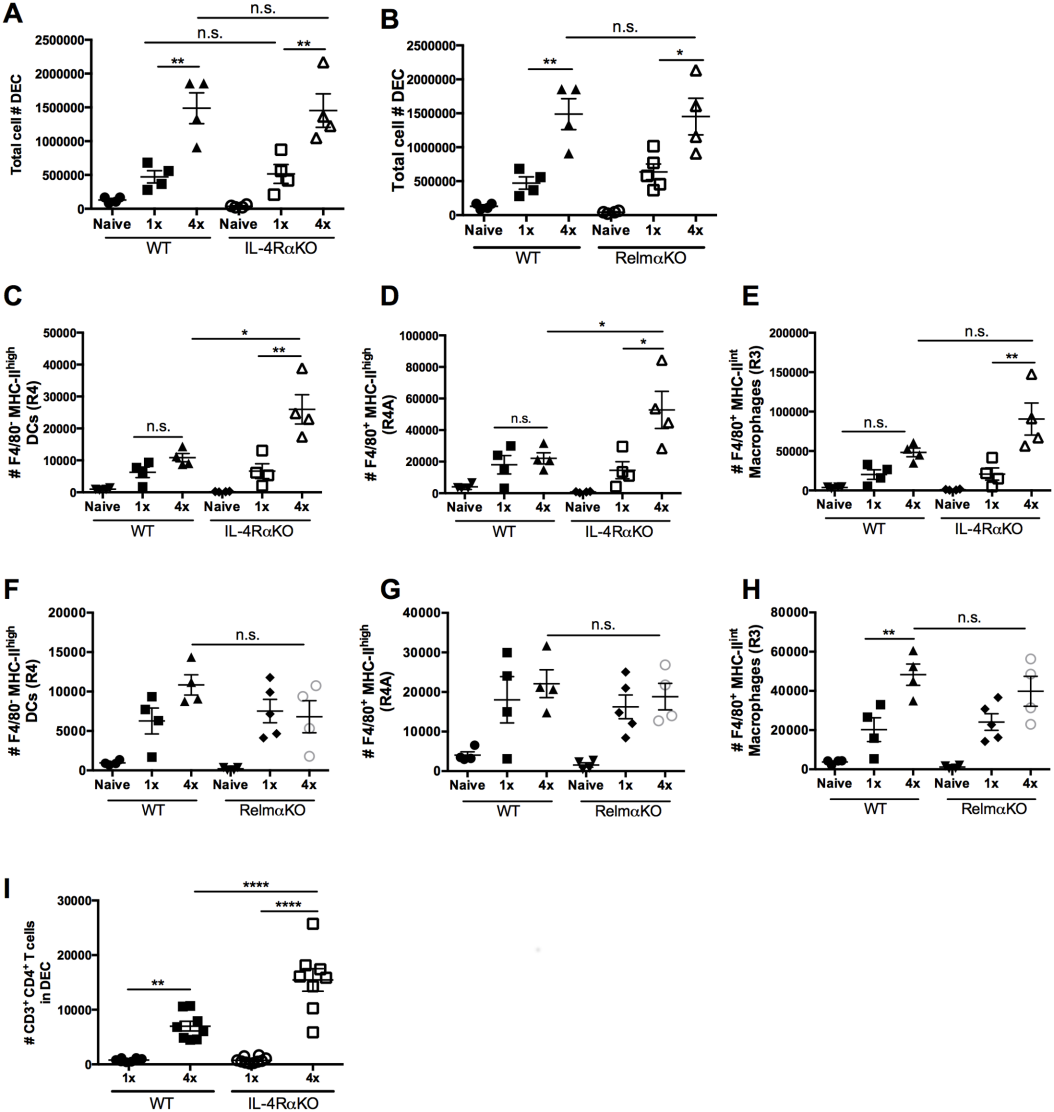
The absence of IL-4Rα and Relmα affects the phenotype of mononuclear phagocytes recovered from the skin. Total number of viable DEC recovered from pinnae of WT and **A.** IL-4RαKO, and **B**. RelmαKO mice. Absolute numbers of **C & F.** F4/80^-^MHC-II^high^ DCs (R4), **D & G.** F4/80^+^MHC-II^high^ tissue resident macrophages (R4A), and **E & H.** F4/80^+^MHC-II^int^ macrophages (R3) in 1x and 4x infected WT, IL-4RαKO and RelmαKO mice. **I.** Absolute numbers of CD3^+^CD4^+^ T cells in the DEC of 1x and 4x infected WT and IL-4RαKO mice. Symbols are values for individual mice; horizontal bars are means ±SEM (n = 4–5 mice per group). n.s. denotes ‘not significant’ p>0.05; * = p<0.05; ** = p<0.01; *** = p<0.001; **** = p<0.0001 as determined by ANOVA and Tukey’s post-test analysis, or Student’s t test.

As expected, significantly greater numbers of DEC were recovered from 4x WT compared to 1x WT mice (Fig 3A and 3B; p<0.01), although as with pinnae thickness, DEC numbers in 1x and 4x infected IL-4RαKO mice were similar compared to their WT cohorts (Fig 3A; p>0.05). The number of DEC recovered from pinnae biopsies of 4x RelmαKO compared to 4x WT mice was also not significantly different (Fig 3B; p>0.05).

The abundance of F4/80^+^ antigen presenting cells may be critical to the development of CD4^+^ T cell hypo-responsiveness, particularly as it has been shown that F4/80^+^ alternatively activated macrophages depress CD4^+^ cell responses following infection with filarial parasites [29]. Here, we show there were no significant changes in the number of F4/80^-^MHC-II^high^ (R4), F4/80^+^MHC-II^high^ (R4A), or F4/80^+^MHC-II^int^ (R3) cells in 4x WT compared to 1x WT mice (Fig 3C–3E; all p>0.05). However, in the absence of IL-4Rα signaling, there were large increases in the numbers of these three cell types in 4x IL-4RαKO compared to 1x IL-4RαKO mice (Fig 3C–3E; p<0.05–0.01). Moreover, the number of R4 and R4A cells, which express high levels of MHC-II, were significantly greater in 4x IL-4RαKO than in 4x WT cohorts (Fig 3C and 3D; p<0.05–0.01), although the change in the number of R3 macrophages which express intermediate levels of MHC-II in 4x IL-4RαKO and 4x WT mice was not statistically different (Fig 3E; p>0.05). In RelmαKO mice, the numbers of R3, R4 and R4A DEC were similar to those in WT cohorts, and there was no significant difference in the number of these two cell types after 4x compared to 1x infection (Fig 3F–3H; p>0.05).

Increased numbers of MHC-II^high^ R4 and R4A cells in 4x infected IL-4RαKO mice could support a stronger T cell response, both in the skin site of infection and down-stream in the draining lymphoid tissue [30, 31]. This might be particularly relevant when coupled with low levels of IL-10 production in the skin, a cytokine that we recently showed to be fundamental in regulating CD4^+^ T cell numbers [6, 8]. Indeed, in the absence of IL-4Rα signaling, significantly greater numbers of CD3^+^CD4^+^ T cells were present in the skin infection site of 4x IL-4RαKO, compared to 4x WT mice (Fig 3I; p<0.0001). Therefore, we subsequently investigated the changes in the numbers, viability, and/or responsiveness of CD4^+^ cells in the sdLN.

### Increased CD4^+^ cellularity in the sdLN of 4x infected mice in the absence of IL-4Rα and RELMα but no reversal of hypo-responsiveness

**Fig 4:**
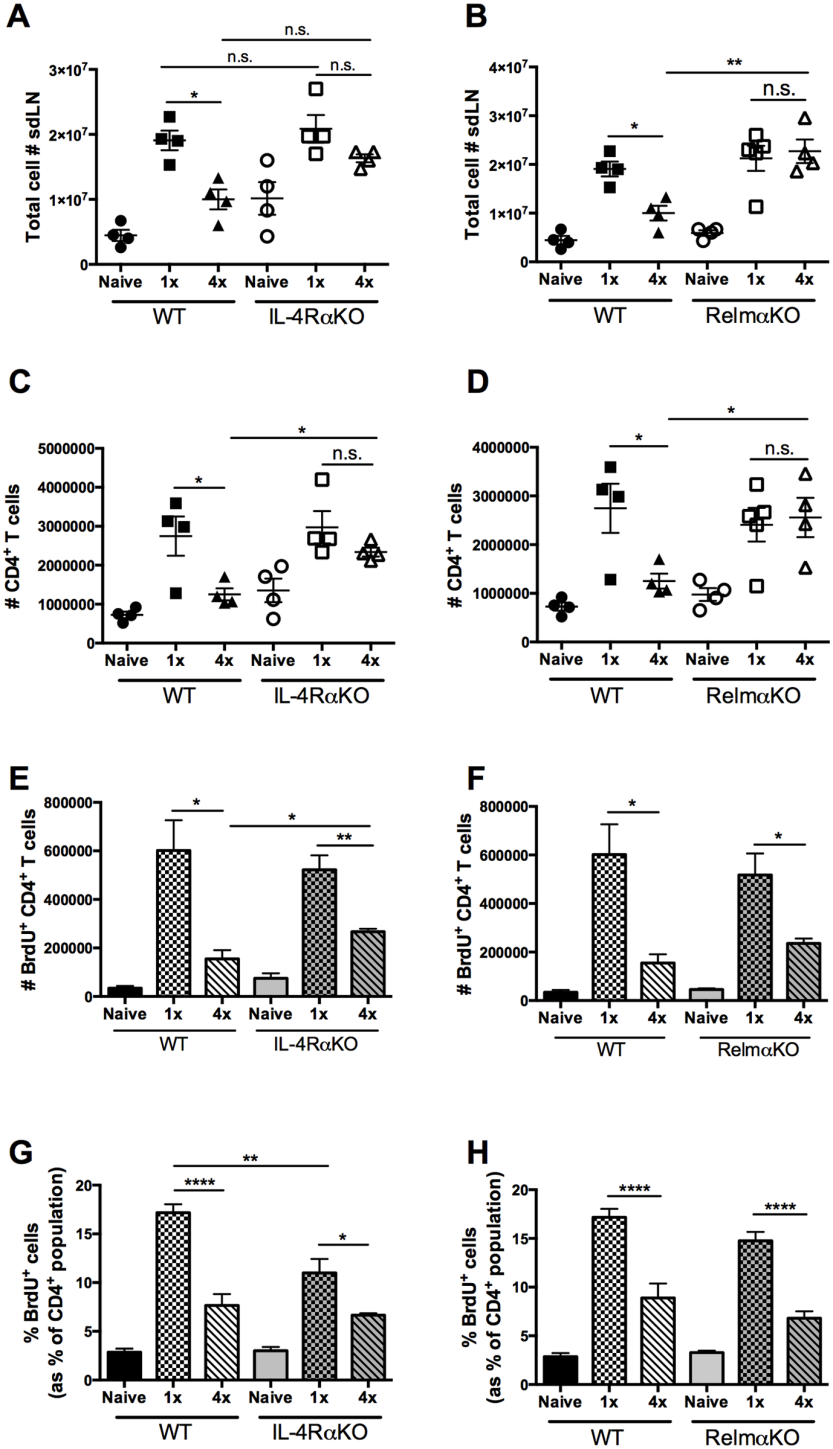
Increased CD4^+^cellularity of sdLN from 4x infected mice in the absence of IL-4Rα and RELMα but not a reversal of CD4^+^ cell hypo-responsiveness. Number of **A** & **B.** total cells, and **C** & **D**. CD4^+^ T cells in the sdLN of naïve, 1x and 4x infected WT, IL-4RαKO, and RelmαKO mice. **E & F** Number of CD4^+^ BrdU^+^ cells, and **G & H.** percentage of CD4^+^ T cells that are BrdU^+^ in the sdLN of naïve, 1x and 4x infected WT mice compared to IL-4RαKO and RelmαKO mice. Symbols are values for individual mice; horizontal bars are means ±SEM (n = 4–5 mice per group). n.s. denotes ‘not significant’ p>0.05; * = p<0.05; ** = p<0.01; *** = p<0.001; **** = p<0.0001 as determined by ANOVA and Tukey’s post-test analysis, or Student’s t test.

After 4x infections, the sdLN of WT mice had significantly fewer cells compared to 1x WT mice (Fig 4A; p<0.05). However, in the absence of IL-4Rα, cellularity was increased, resulting in comparable cell numbers between 1x and 4x infected IL-4RαKO mice which were not significantly different (Fig 4A; p>0.05). Similarly, total sdLN cellularity was also increased in 4x RelmαKO compared to 4x WT mice (Fig 4B; p<0.01), resulting in the number of sdLN cells from 4x and 1x RelmαKO mice being not significantly different (Fig 4B; p>0.05).

There were also decreased numbers of CD4^+^ T cells in the sdLN of 4x compared to 1x WT mice (Fig 4C; p<0.05), although the number of CD4^+^ T cells in 4x compared to 1x IL-4Rα KO mice was not significantly reduced (Fig 4C; p>0.05). Therefore, in comparison there were significantly more CD4^+^ T cells in 4x IL-4RαKO than in their 4x WT counterparts (Fig 4C; p<0.05). The numbers of CD4^+^ T cells in the sdLN of 1x and 4x RelmαKO mice was also similar (Fig 4D; p>0.05) leading to significantly greater numbers of CD4^+^ T cells being detected in the sdLN of 4x RelmαKO compared to 4x WT mice (Fig 4D; p<0.05). This showed that the absence of either IL-4Rα, or RELMα, leads to increased number of CD4^+^ T cells in the sdLN following repeated infection comparable to the levels seen in 1x mice.

It was proposed that the increased number of CD4^+^ T cells in sdLN of 4x IL-4Rα and 4x RelmαKO mice could be due to a reversal in the development of hypo-responsiveness normally seen in 4x WT mice. In fact, the total number of BrdU^+^CD4^+^ cells was slightly greater in 4x IL-4RαKO mice compared to the 4x WT cohort group (Fig 4E; p<0.05), although the number in 4x RelmαKO versus 4x WT mice was not significantly different (Fig 4F; p>0.05). While ~15–20% of the CD4^+^ T cells in the sdLN from 1x WT mice were BrdU^+^ and had therefore proliferated *in vivo*, only ~7% from 4x WT mice were BrdU^+^ (Fig 4G; p<0.0001) confirming the establishment of CD4^+^ T cell hypo-responsiveness. However, although there were a slightly greater number of BrdU^+^ cells in 4x IL-4RαKO mice (Fig 4E), the proportion of BrdU^+^ cells in 4x IL-4RαKO mice remained significantly lower than in 1x IL-4RαKO mice (Fig 4G; p<0.05) demonstrating that the absence of IL-4Rα does not restore proliferation of CD4^+^ T cells in the sdLN of 4x mice to the levels seen in 1x WT mice. A similar situation was observed in 4x RelmαKO mice, where the proportions of BrdU^+^ cells in 4x RelmαKO compared to 4x WT mice were not significantly different (Fig 4H; p>0.05) and both exhibited CD4 T cell hypo-responsiveness *in vivo* compared to their 1x cohorts (Fig 4H; p<0.0001). This shows that the absence of RELMα also does not restore CD4^+^ T cell proliferation in the sdLN after repeated exposures of the skin to *S*. *mansoni* cercariae.

### Expression of Fas/FasL and viability of CD4^+^ cells in the sdLN depends upon the presence of IL-4Rα

**Fig 5:**
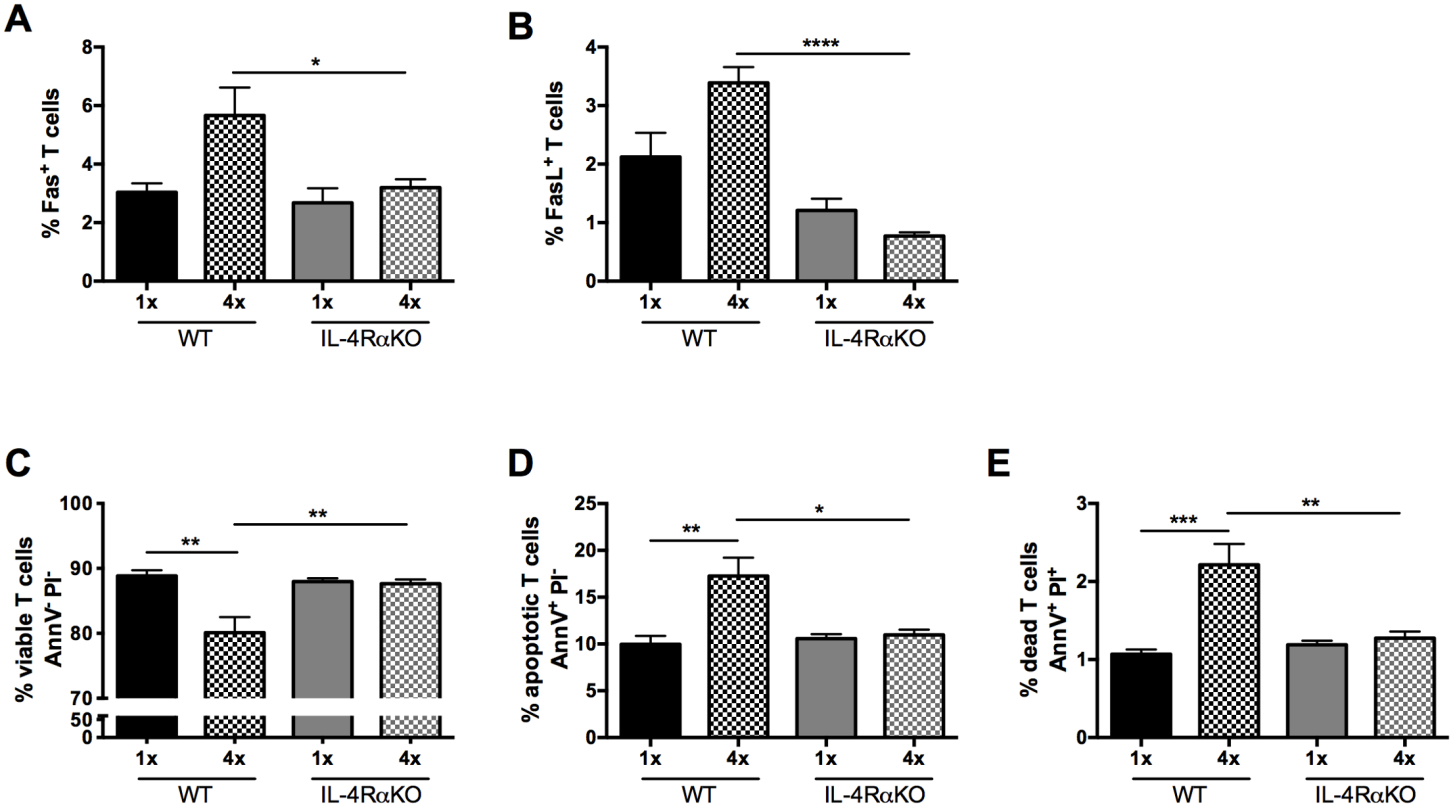
Expression of Fas/FasL and viability of CD4^+^ T cells in the sdLN is enhanced in the absence of IL-4Rα. Percentage of CD4^+^ T cells that are **A.** Fas^+^ and **B.** FasL^+^ in 1x and 4x infected WT compared to IL-4RαKO mice. **C-E** Percentage of viable (AnnV^-^PI^-^), apoptotic (AnnV^+^PI^-^) and dead (AnnV^+^PI^+^) CD4^+^ T cells in the sdLN of 1x and 4x infected mice as determined by an AnnexinV assay. n = 3–4 mice per group; n.s. denotes ‘not significant’ p>0.05; * = p<0.05; ** = p<0.01; **** = p<0.0001 as determined by ANOVA and Tukey’s post-test analysis.

Previously, we reported that CD4^+^ T cell hypo-responsiveness in the sdLN observed after repeated infection is dependent on IL-10 [8]. This was due to increased CD4^+^ T cell activation accompanied by decreased death and apoptosis of the CD4^+^ T cell population in the sdLN. In the current study, we show that both IL-4RαKO and RelmαKO mice had decreased IL-10 production in the skin and increased cellularity in the sdLN after 4x infection. As RELMα expression is dependent on IL-4 signaling [32], we restricted further analysis of the CD4^+^ cell population to cells in the sdLN of 1x versus 4x IL-4RαKO mice. We observed that surface protein expression of both Fas and FasL increased in CD4^+^ T cells recovered from 4x WT compared to 1x WT mice but was significantly decreased in 4x IL-4RαKO compared to 4x WT mice (Fig 5A and 5B; p<0.05 and p<0.0001). In addition, whereas in WT mice, there was a significant decrease in the proportions of AnnV^-^PI^-^ viable CD4^+^ T cells in 4x compared to 1x mice (Fig 5C; p<0.01), the absence of IL-4Rα signaling resulted in significantly greater proportions of AnnV^-^PI^-^ viable CD4^+^ T cells in 4x IL-4RαKO compared to 4x WT mice (Fig 5C; p<0.01). This caused there to be no significant difference between the viability of CD4^+^ T cells from 4x IL-4RαKO mice compared to either 1x WT, or 1x IL-4RαKO mice (Fig 5C; p>0.05). The increase in the proportion of AnnV^-^PI^-^ viable CD4^+^ T cells in 4x IL-4RαKO mice was accompanied by the detection of significantly fewer AnnV^+^PI^-^ apoptotic CD4^+^ T cells (Fig 5D; p<0.05), as well as fewer AnnV^+^PI^+^ dead CD4^+^ T cells, compared to 4x WT mice (Fig 5E; p<0.01). Thus, it appears that a reduction in IL-4Rα signaling facilitates CD4^+^ T cell survival. This increased survival could explain why the number of CD4^+^ T cells in the sdLN of 4x IL-4RαKO mice is not significantly reduced after repeated exposure to schistosome cercariae which contrasts with the situation in 4x WT mice where there is a significant reduction in number of CD4^+^ T cells when compared to their 1x WT counterparts.

## Discussion

Here we demonstrate that three discrete mononuclear phagocyte cell populations were present in DEC recovered from the skin infection site of mice exposed to repeated doses of schistosome cercariae. Moreover, all three populations exhibited an alternatively activated phenotype as judged by microarray analysis of DEC. For example, *Retnla* (encoding RELMα) which is a key marker of alternatively activated macrophages [33, 34] was one of the most highly up-regulated genes in 4x compared to 1x DCs, tissue macrophages, and macrophages. Several other genes also linked to alternative activation were up-regulated in the three discrete 4x DEC populations including *chi3l3* and *chi3l4* (encoding chitinase-like molecules YM-1 and YM-2 [12, 33]). The gene for IL-4 was also up-regulated in the macrophage DEC population after repeated infections, and IL-4 has been shown to be produced by alternatively activated/type-II macrophages using both human and mouse cells [35, 36]. Other up-regulated genes linked to alternative activation were *mrc1* and *cd209* which encode the C-type lectins for the mannose receptor CD206 and DC-SIGN CD209 [9], whilst expression of *cd163* encoding the scavenger receptor CD163 when used in combination with *chi3l3* and *Retnla* [11, 12, 37] also supports the identification of the three discrete 4x DEC populations as being alternatively activated. Conversely, *Arg1* which we had previously reported to be up-regulated in DEC from 4x mice [4], was not >2 fold up/down-regulated in the any of the 3 sorted cell populations, whilst *Clec10a* of the C-type lectin/C-type lectin-like domain (CTL/CTLD) superfamily, associated with alternative activation [9] was only 2.2 fold up-regulated in the DC population. On the other hand, wound healing associated genes such as *igf-1* encoding Insulin-like growth factor 1 suggested to be involved in resolving tissue damage following helminth infection [38], and *fn1* encoding fibronectin produced during the resolution of tissue damage [39] were both highly up-regulated. This provides evidence supporting an evolutionary link between alternative activation in mononuclear phagocytes and the resolution of tissue damage caused by helminth infection [16, 17, 38]. Indeed, we have shown that schistosome cercariae cause significant tissue disruption as they invade through the skin [3], and therefore tissue repair of the skin following repeated exposure to invasive schistosome cercariae should be expected.

The discrete DEC mononuclear phagocyte populations all express genes typically associated with alternative activation [9–12]. This provides a more in-depth analysis of DEC from 4x mice in which mononuclear phagocytes appeared to switch from classically-activated to alternatively-activated commensurate with up-regulated mRNA transcripts for Ym1 and RELMα but only low levels of iNOS and IFNγ [4]. Nevertheless, whilst the R4, R4A, and R3 mononuclear phagocytes all appear to be alternatively activated, subtle qualitative differences in the expression of specific genes between the three DEC populations underline the likely heterogeneity/plasticity of these mononuclear phagocytes which may have different and/or overlapping functional roles *in vivo* [40–42].

In the context of repeat infection of the skin with *S*. *mansoni* cercariae, DEC recovered from the site of infection had a pronounced increase in the expression of RELMα but its absence had no effect on the extent of skin inflammation (i.e. pinnae thickness), or on the numbers of mononuclear phagocyte DEC populations recovered from the skin infection site (Fig 3F–3H). The absence of RELMα did however result in a slight reduction in IL-10 production in the skin alongside an increase in the numbers of CD4^+^ cells in the sdLN such that they were as abundant as in 1x RELMαKO mice and were more numerous than in 4x WT cohorts. However, the absence of RELMα did not affect the development of CD4^+^ T cell hypo-responsiveness in the sdLN of 4x mice. In contrast, a separate study showed that RELMα KO mice infected with *Nippostrongylus brasiliensis* exhibit enhanced intestinal and pulmonary pathology accompanied by expulsion of the parasite indicating a regulatory role for RELMα by dampening normally protective strong Th2-dependent responses [32, 43]. Moreover, pulmonary immune granulomas to injected *S*. *mansoni* eggs were enhanced in the absence of RELMα [44], as were hepatic immune granulomas formed at the chronic phase of schistosome infection [32]. Nonetheless, these previous studies were performed when the immune response was skewed towards a Th2 phenotype [21]. In contrast, the response investigated in our study occurs at an early phase of infection, prior to egg-induced Th2 biased immunopathology, and is accompanied by cytokines with a mixed Th1 and Th2 type response [4]. Therefore, RELMα may only have a regulatory role during strong Th2 responses during the chronic phase of infection and we suggest that it has less of a role when the immune response has a mixed Th1/Th2 phenotype.

Signaling through IL-4Rα is well defined as being important in the development of alternative activation [10, 12] and in the maintenance of Th2 responses [45]. Whilst the absence of IL-4Rα did not have an impact on inflammation at the skin site of infection, nor on the number of DEC, its absence significantly increased the numbers of cells expressing high levels of MHC-II. In addition, increased release of pro-inflammatory IL-12p40 in the absence of IL-4Rα was accompanied by decreased levels of regulatory IL-10. Therefore, we considered it possible that the increased numbers of cells with antigen presenting potential in the skin might lead to enhanced CD4^+^ cell activity downstream in the sdLN. However, whilst the absence of IL-4Rα resulted in increased numbers of CD4^+^ cells in the skin infection site and the sdLN, the cells in the sdLN remained hypo-responsive *in vivo* to stimulation with parasite antigen similar to 4x WT mice. The use of cell specific gene deficient mice, such as those expressing a Cre recombinase from the lysozyme M-encoding locus, which has been widely used in the context of IL-4Rα function in macrophages and in the context of schistosome infection [22], would have been desirable to fully interrogate the role of particular genes on mononuclear phagocytes. However, such mice were not available during the current project. In addition, it has been reported that Il4rα excision in these mice is incomplete during inflammatory conditions [46], raising doubts about interpretation of data obtained using these mice and suggests that alternative cell specific gene animals should be sought.

Further analysis of CD4^+^ cells in the sdLN revealed that there was a significant elevation in the expression of Fas and FasL on the CD4^+^ T cells in the sdLN in 4x infected WT mice, whereas in the absence of IL-4Rα the expression levels of these two molecules was not elevated and was not different from those in 1x IL-4Rα mice. We also showed that skin biopsies from 4x IL-4RαKO mice, in contrast to 4x WT cohorts, released negligible quantities of IL-10 suggesting a possible role for IL-4Rα in the promotion of IL-10 production. Indeed, IL-4Rα signaling is required for the production of IL-10 derived from Th2 cells following infection with *N*. *brasiliensis*, thereby resulting in increased levels of regulation via IL-10 [47], although others show that IL-10 production following chronic schistosome infection can be IL-4Rα-independent [48]. Here, in our study we found that IL-10 production in the skin was significantly reduced in the absence of IL-4Rα, potentially resulting in decreased regulation via IL-10.

Several studies have identified a link between IL-10 and Fas/FasL expression. For example in systemic lupus erythematosus (SLE), IL-10 is directly able to induce the expression of Fas on the surface of T cells resulting in increased levels of apoptosis [49–51]. Moreover, we recently showed that in IL-10KO mice, there was reduced Fas expression on CD4^+^ T cells in the sdLN following repeated schistosome infection which led to a reduction in CD4^+^ T cell death in the sdLN and consequently may have contributed to the alleviation of CD4^+^ T cell hypo-responsiveness in the absence of IL-10 [8]. Here, we suggest a novel mechanism for the regulation of the immune response through IL-4Rα, which impacts both IL-10 production and antigen presenting cells numbers, which would subsequently regulate Fas and FasL expression on CD4^+^ T cells in the sdLN of 4x infected mice. Thus, IL-4Rα signaling results in increased IL-10 production, increased levels of apoptotic and/or dead T cells in 4x mice and a dampening of the immune response. This link between IL-4Rα and IL-10 and Fas/FasL-induced apoptosis could be a potential novel mechanism through which IL-4Rα regulates the immune system.
